# Advancing precision medicine therapeutics for Parkinson’s utilizing a shared quantitative systems pharmacology model and framework

**DOI:** 10.3389/fsysb.2024.1351555

**Published:** 2024-03-08

**Authors:** Christopher Denaro, Diane Stephenson, Martijn L. T. M. Müller, Benedetto Piccoli, Karim Azer

**Affiliations:** ^1^ Center for Computational and Integrative Biology, Rutgers University, Camden, NJ, United States; ^2^ Critical Path Institute, Tucson, AZ, United States; ^3^ Department of Mathematical Sciences, Rutgers University—Camden, Camden, NJ, United States; ^4^ Axcella, Cambridge, MA, United States

**Keywords:** quantitative systems pharmacology, precision medicine, translational research, disease modifying therapy, genetics, biomarkers, Parkinson

## Abstract

A rich pipeline of therapeutic candidates is advancing for Parkinson’s disease, many of which are targeting the underlying pathophysiology of disease. Emerging evidence grounded in novel genetics and biomarker discoveries is illuminating the true promise of precision medicine-based therapeutic strategies for PD. There has been a growing effort to investigate disease-modifying therapies by designing clinical trials for genetic forms of PD - providing a clearer link to underlying pathophysiology. Leading candidate genes based on human genetic findings that are under active investigation in an array of basic and translational models include SNCA, LRRK2, and GBA. Broad investigations across mechanistic models show that these genes signal through common molecular pathways, namely, autosomal lysosomal pathways, inflammation and mitochondrial function. Therapeutic clinical trials to date based on genetically defined targets have not yet achieved approvals; however, much is to be learned from such pioneering trials. Fundamental principles of drug development that include proof of pharmacology in target tissue are critical to have confidence in advancing such precision-based therapies. There is a clear need for downstream biomarkers of leading candidate therapies to demonstrate proof of mechanism. The current regulatory landscape is poised and primed to support translational modeling strategies for the effective advancement of PD disease-modifying therapeutic candidates. A convergence of rich complex data that is available, the regulatory framework of model informed drug development (MIDD), and the new biological integrated staging frameworks when combined are collectively setting the stage for advancing new approaches in PD to accelerate progress. This perspective review highlights the potential of quantitative systems pharmacology (QSP) modeling in contributing to the field and hastening the pace of progress in advancing collaborative approaches for urgently needed PD disease-modifying treatments.

## 1 Introduction: Key scientific and regulatory advances and enablers, development challenges and opportunities for precision medicine development in PD

There has been significant advancement and momentum in fulfilling medical unmet needs for patients with neurodegenerative diseases. Technological and methodological innovations have enhanced the quantitative understanding of brain physiology and pathophysiology and the effects of therapeutic interventions. Progress in genetics and genomics, catalyzed by the human genome project and advances in science and technology as applied to the nervous system have paved the way for important discoveries with the linkage of genetic variants to the underlying pathophysiology of neurodegenerative diseases. Moreover, there have been tremendous regulatory innovations to accelerate the pace of drug and vaccine approvals, as exemplified and catapulted by the enormous medical needs ensuing from the COVID-19 pandemic. While drug developers have worked in record time to advance innovative vaccines, regulators around the world have provided the appropriate paths for accelerated emergency authorizations and ultimately approvals in the face of a global pandemic. The pandemic served as a catalyst to reevaluate processes that were archaic and inefficient, which paved the way for new innovative approaches to clinical trial design and execution. In the case of neurodegenerative diseases, scientific discoveries coupled with regulatory advances have upended many years of unsuccessful attempts at therapeutic intervention and have already enabled important approvals in medicine. Recent examples of drug approvals such as Nesinersen for Spinal muscular atrophy, Tofersen for ALD-SOD1, Lecanemab for Alzheimer’s disease, and Omaveloxolone for Friedreich’s ataxia are laying the groundwork for other neurodegenerative diseases at an astonishing pace (Hoy, 2021; Saini and Chawla, 2023; van Dyck et al., 2023; Vitek et al., 2023).

Parkinson’s Disease (PD) is a debilitating progressive neurodegenerative disease that affects nearly 9.5 million people worldwide (GBD, 2016 Parkinson’s Disease Collaborators, 2018). More than 90,000 people in the U.S. are diagnosed each year, and prevalence is rapidly growing over the next several years (“Estimation of the 2020 Global Population of Parkinson’s Disease PD,”2023; Ou et al., 2021). The burden of PD on the lives of people affected by the disease is devastating, and currently, approved dopaminergic medications that treat motor symptoms are effective in improving motor symptoms yet lose efficacy as the disease progresses. A complex array of nonmotor symptoms are not effectively treated despite the burden they play on the overall quality of life (Hermanowicz et al., 2019). There are two broad categories of therapies for PD, namely, symptom-modifying therapies (SMTs) and disease-modifying therapies (DMTs) (Sardi et al., 2018; Ntetsika et al., 2021; McFarthing et al., 2022). As the names suggest, SMTs focus on relieving the motor symptoms of PD, such as bradykinesia and tremors; whereas DMTs aim to slow or halt disease progression. Although currently approved treatments can dramatically improve the lives of people with PD in the first few years of disease diagnosis, they do not effectively treat the disabling nonmotor symptoms, nor do they address the underlying causes of the disease or the inevitable disease progression. There has been an expanding focus on developing DMTs for PD prompted by emerging advances in research into the underlying biology and genetics of the disease. The 2023 clinical pipeline publication of PD therapeutics reports that there are more than 130 clinical trials for PD, of which 65 are investigating DMTs (McFarthing et al., 2022). Despite the increase of focus on DMTs and advances in precision medicine therapeutics, there are many challenges to overcome in order to achieve approval of disease-modifying therapies.

There is an extraordinarily high rate of failures in clinical trials of DMTs across neurodegenerative diseases, including PD (Mortberg et al., 2022). Challenges are multifactorial and span a range of issues including 1) the poor translatability of preclinical and animal models in predicting drug efficacy for neurodegenerative diseases (Dawson et al., 2018), 2) the long duration of time from disease onset to manifestation of clinical symptoms in humans to objectively measure disease progression in trials (McGhee et al., 2016; Cummings, 2017), 3) the profound neurodegeneration that is present at the time of clinical diagnosis (Jellinger, 2009) and 4) the fact that CNS disorders are localized to a body compartment that is not readily accessible for obtaining tissue or biofluid samples poses a hurdle for successful noninvasive measurement of target engagement in humans. Such examples pose barriers for implementation of precision medicine in humans that are quite distinct from conditions such as cardiovascular, oncology and immunology that have paved the way for successful drug approvals over the past decades. Transformation is taking place given that 2023 was a year of landmark approvals of neurology products with 9 approvals, second only to oncology indications. Several of the neurology approvals were of disease modifying therapeutics in disorders with unprecedented treatments (e.g., Frierdrichs ataxia) (Mullard, 2024).

There is growing evidence that early intervention holds the most promise for disease modification and an urgent need to advance trials targeting prevention. A recent study reported the negative results of drug trials in neurodegenerative diseases over the past 2 decades (Mortberg et al., 2022). A systematic evaluation of clinical trial registration data was conducted to analyze the characteristics of trials in four major neurodegenerative diseases (AD, PD, ALS, and FTD). Of the 3,238 neurodegenerative disease clinical trials evaluated, only 2.7% of trials were investigated in pre-symptomatic individuals. A total of only sixteen novel targets tested in drug trials were based on genetically supported therapeutic hypotheses, thus representing only a small, non-increasing fraction of trials. Moreover, the mean lag from genetic association to first trial was 13 years. The authors concluded that additional investment in well-powered, well-controlled trials at earlier disease stages may be needed to realize targets that hold potential for disease modification and prevention. New initiatives targeting gene based therapeutics, biological staging of disease and target populations prior to onset of clinical symptoms are emerging at a rapid pace in neurodegenerative diseases including in PD (Crotty et al., 2022; Foltynie et al., 2023)with strong support from the broader community including at risk individuals (Keavney et al., 2023). Enabling early detection diagnostic biomarkers are critical to support the earlier initiation of disease modifying therapy, and the development of these early biomarkers rests on longitudinal studies that enable the discovery and utility of these markers with the aid of artificial intelligence and systems biology methodologies.

In PD, a diversity of factors have been proposed to contribute to the challenges in the success of DMTs, such as heterogeneity of clinical and pathologic endophenotypes, unpredictable placebo responses, long duration of trials, and challenges in translation of animal models (Espay et al., 2020; Devos et al., 2021). One of the many contributing factors is the lack of validated biomarkers for quantifying disease progression in disease-modifying trials. Moreover, in a review of about 121 trials in neurodegenerative diseases, less than half reported the use of central biomarkers, and a little more than half of trials included at least one target occupancy/activation biomarker (Vissers et al., 2021). However, there is reason for optimism in this area given the rapid progress being made in the development of biomarkers as drug development tools for Neurodegenerative diseases (Marks et al., 2021; Qi et al., 2023) and in PD specifically. Recent progress in biomarkers for PD has advanced with the identification of disease hallmark signatures that were previously only detected at autopsy to confirm pathologic diagnosis. Alpha-synuclein, the key protein of Lewy Bodies, can now be assessed with exquisitely high specificity and sensitivity *in vivo* nearly a decade prior to the onset of clinical symptoms (Siderowf et al., 2023). Additional new biomarkers that are assessed by neuroimaging and biofluid methodologies are also advancing at a rapid rate. The concept of multimodal biomarkers is also taking hold as a key to success in identifying the onset and trajectories of heterogeneous complex diseases (Carter et al., 2023).

Biomarkers are powerful tools to enable confidence in decision-making along the drug development lifecycle for diverse applications, including diagnosis, patient stratification, pharmacodynamic response, and surrogates of efficacy. Importantly, the rate of drug approvals across diseases is known to increase when biomarkers are utilized in trials (Gromova et al., 2020). The use of pharmacodynamic biomarkers to demonstrate target engagement in early clinical development is key to assuring proof of pharmacology in target tissue. The concept of three pillars of drug development was outlined by industry leaders more than a decade ago, where the roadmap requires demonstrating target engagement as essential before advancing new candidates to subsequent stages (Morgan et al., 2012). Demonstration of proof of concept is challenging in drugs that target nervous system disorders due to the need to show target engagement of drugs that cross the blood-brain barrier and show dose-responsive signals that can be measured in a central compartment. Mechanistic studies can be applied in translational models in ways that address the valley of death, and such studies clearly enhance confidence in dose selection and confidence in mechanism in unique ways. A review of the reported rate of mechanistic and physiological response biomarkers across neurodegenerative disease trials of DMTs showed that only 54% of trials used mechanistic (target occupancy or activation) biomarkers to demonstrate target engagement in humans (Vissers et al., 2021). Thus, there is a need to improve proof of mechanism and target engagement for all PD, particularly DMTs that are in the pipeline for PD.

There has been significant progress in Parkinson’s disease, with the identification of genetically defined targets, advancement of biomarkers of the disease, and, more recently, new efforts to outline the biological staging framework of the disease. The current understanding of the pathophysiology of PD has evolved significantly based on advances in human molecular genetics. Such findings are now elucidating specific molecularly defined therapeutic targets for intervention and common pathways amongst distinct candidate genes (Sardi et al., 2018). These advances are now leading to refining the traditional syndromic definition of PD to enable precision medicine therapeutic strategies (Schneider et al., 2020). The rapid evolution of clinical trials targeting known risk genes has evolved this past decade with key targets that focus on LRKK2, GBA, and synuclein (SNCA) (Merchant et al., 2019; Senkevich et al., 2021; Jasutkar et al., 2022; Magalhães and Lashuel, 2022; Taymans et al., 2023). Leading clinical trials have not been successful to date; yet, there is much to learn from these pioneering studies (Lang et al., 2022; Pagano et al., 2022; Giladi et al., 2023).

With the discovery of genetically defined targets for Parkinson’s disease, biomarker research, and advancements in translational modeling strategies, we have a unique opportunity to optimize and de-risk the translation of candidates to the clinic by outlining the path for successful clinical proof of concept. By bringing together improved mechanistic disease knowledge and link to genetics, novel biomarkers, advanced modeling tools and frameworks, and innovative regulatory advances and pathways, we hold the unique opportunity as a community to set an accelerated pace towards an approved therapy for Parkinson’s. In this perspective, we outline this call to action for public and private entities and patients for a roadmap to success in collaboratively advancing disease-modifying therapies for PD.

## 2 Review of the genetic bases and associated pathways of PD and implications for biomarker development and disease stratification

*Genetics and pathways.* The etiology of PD is complex and multifactorial with contributions that include environmental exposures, polygenic inheritance, and gene environment interactions. Genetic forms of PD that are monogenic in nature are relatively rare overall (Klein et al., 2018). LRKK2, GBA, and SNCA have been key genes of focus including their role as novel therapeutic candidates for disease modification. Human molecular genetics are paving the way with Genome-wide association studies (GWAS) identifying several gene mutations that confer a risk of developing PD (Billingsley et al., 2023; Rizig et al., 2023). Risk-associated genes may have different cellular roles, ranging from energy production to protein degradation. Some common risk-associated genes include LRRK2, PARK7, PINK1, PRKN, SNCA, and GBA, as described in (Blauwendraat et al., 2020). Such genetic findings represent a unique window into the underlying pathophysiology of disease-causing mutations in specific target genes. Many reviews have been published that represent compilation of the genetics of PD (e.g., Singleton; Greenamyre, Alcalay) and this is out of scope for this current perspective review. However, it is striking to see the rapid progress in defining downstream signaling pathways that are linked to specific candidate genes. Furthermore, emerging data demonstrate that distinct candidate target genes can signal through common molecular pathways (Navarro et al., 2021; Kaiser et al., 2023; Gialluisi et al., 2021; Bandres-Ciga et al., 2020; Vollstedt et al., 2023). Key mechanisms that are shared amongst distinct candidate genes include autosomal lysosomal function, mitochondrial integrity and function as well as inflammatory signaling pathways (Sardi et al., 2018; Senkevich and Gan-Or, 2020; Gao et al., 2022; Muñoz-Delgado et al., 2023).

The concept of precision medicine based strategies for PD originated soon after the discovery of the link to LRKK2 gene responsible for genetic PD as defined by PARK8 locus. Nearly 20 years later, a long journey of research led by academics and industry has been actively pursuing therapies based on a toxic gain of function hypothesis for LRRK2. Despite a large diversity of model systems and translational research approaches aimed at understanding the fundamental biology, at present, the field is still aiming to define key questions that are key to address. In advancing successful therapies based on inhibition of LRKK2, key questions remain. Examples include the need to gain an understanding of why distinct LRKK2 mutations lead to pathology that differs from idiopathic PD (Rajput et al., 2006; Hasegawa et al., 2009), to determine whether targeting LRKK2 in the periphery would be beneficial or detrimental based on safety (Taymans et al., 2023), and why disease progression of LRKK2 gene carriers is unexpectedly slower than idiopathic PD in both motor and nonmotor symptoms (Ahamadi et al., 2019; 2022).

The progress in PD pathophysiology has led to discovery of novel biomarkers that have been catalyzed by the availability of large prospective natural history datasets that contain detailed clinical, imaging, genetic and fluid biomarkers. Examples of novel biomarkers that have emerged recently include synuclein as assessed by seeding amplification assays (Siderowf et al., 2023; Concha-Marambio et al., 2023), LRKK2 mediators (Vissers et al., 2023) and new mitochondrial activity (Rui et al., 2023) biofluid biomarkers. Such biomarkers hold potential for use in patient stratification as well as to document biological effects of candidate drugs and proof of pharmacology.

Biological staging of disease represents a paradigm shift in catalyzing drug development by targeting stages of the disease prior to onset of clinical symptoms. A novel biological staging framework for Neuronal Synuclein Disease (NDS) grounded in innovative advances in biomarkers and genetics has been proposed (Chahine et al., 2023). NSD is defined by the presence of pathologic n-asyn assessed by a validated *in vivo* biomarker and the ultimate presence of dopaminergic neuronal dysfunction via DAT SPECT neuroimaging. This biologic definition is independent of the presence of clinical features, or if present, of the specific clinical syndrome. Critical success factors for biological staging of disease are the ability to appropriately stage the disease process through the integration of translational platforms, clinical outcome assessment tools, biomarkers, genetics, and quantitative solutions to optimize trial design.

## 3 Role and impact of QSP in drug development and opportunity to advance PD therapeutic candidates through clinical proof of concept

**FIGURE 1:**
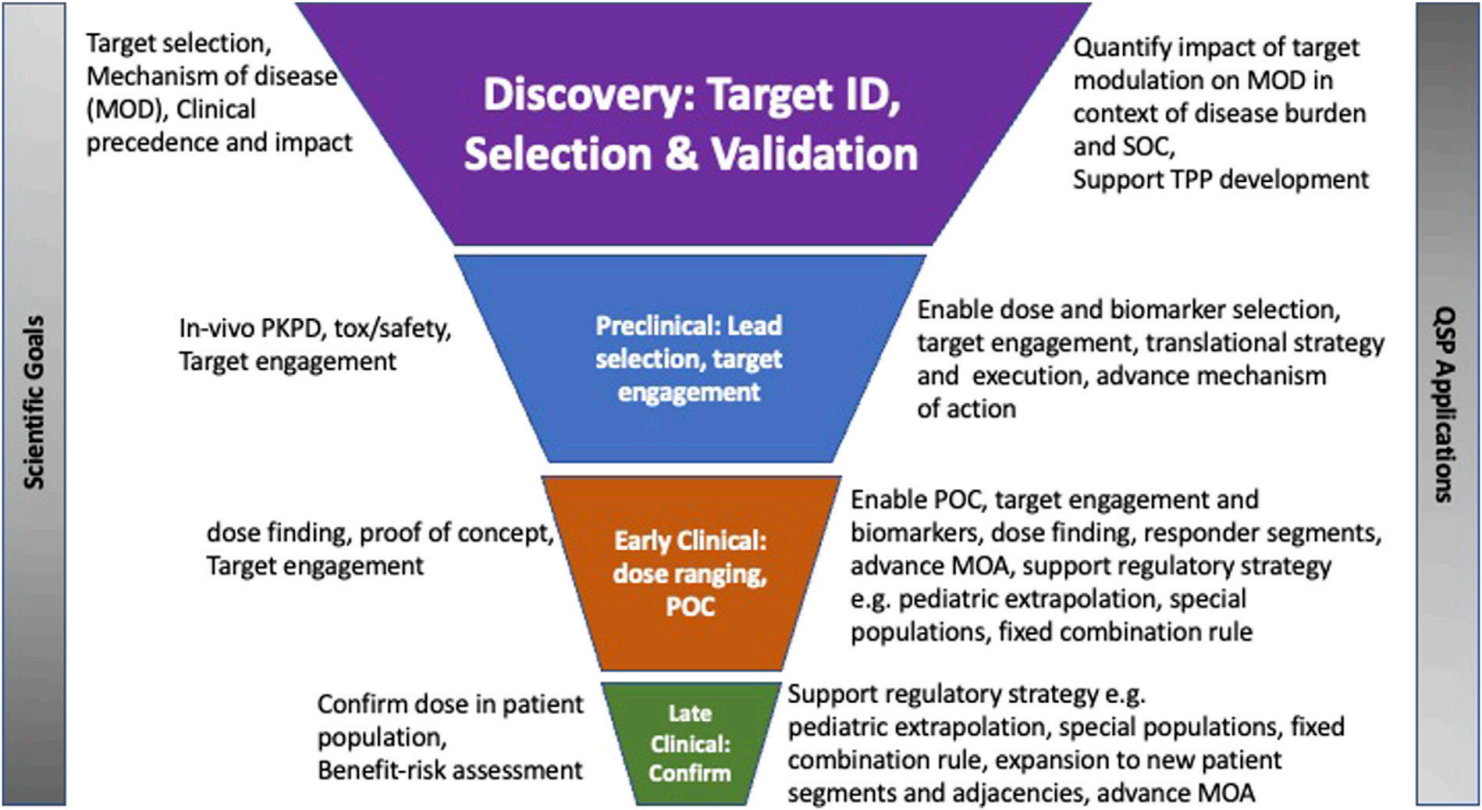
Legend: Application of QSP modeling in drug discovery and development stages Graphical illustration of the role of translational QSP modeling in drug development decision making from target identification to late clinical development.

Quantitative Systems Pharmacology (QSP) is a mechanistic modeling approach that is utilized for the assessment of therapeutic molecular candidates for a disease by linking descriptions of the molecular and cellular mechanisms of the disease and drug to system-wide dynamics, bridging biomarkers and clinical endpoints relevant for the disease (Azer et al., 2021; Barrett and Azer 2023; Gadkar et al., 2016; Balbas-Martinez et al., 2018; Kaddi C. D. et al., 2018; Coletti et al., 2020). There is a chartered translational medicine course and track record, with many published examples where integrating clinical data with biological and pharmacological datasets and encapsulated into a QSP model has enabled the advancement of candidates through the clinic and the refinement or optimization of clinical study design such as biomarkers, or patient selection or dose, elucidation of disease target biology and impact of therapeutic modulation on downstream pathways, as well as QSP platforms that have enabled the prediction, de-risking or characterization of drug induced toxicities such as liver or cardiac toxicities (Figure 1). QSP modeling combines data and knowledge on the mechanisms of disease with drug characteristics to predict biological or clinical changes under pharmacological intervention (Sorger, 2011). While there are a few examples of cross-industry collaborations in building QSP platforms, the bulk of applications and impact have been individual company and portfolio-driven. Given the mechanistic nature of QSP models, the driving hesitation to building cross-company models is the inadvertent sharing of proprietary and competitive intelligence on specific lead molecules. However, data and model-sharing best practices and existing cross-industry collaboration models in QSP and more broadly in systems biology and other disciplines across the industry provide ample evidence that developing and executing precompetitive collaborations, where QSP model structures and system parameters are shared, and proprietary molecule specific attributes remain confidential, can be successful and carry the advantages of accelerated timelines and economies of scale.

**FIGURE 2:**
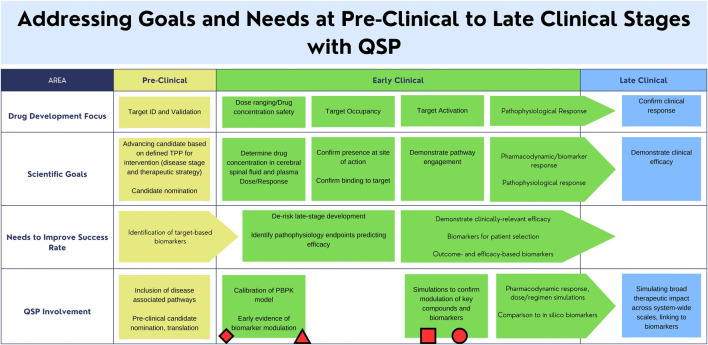
1egend: Areas of clinical development that can be supported by the utilization of a Quantitative Systems Pharmacology (QSP) model. Applications of existing mechanistic models for Alzheimer’s Disease, Tuberculosis, Gaucher Disease, Amyotrophic Lateral Sclerosis, and their relation to the regulatory landscape are indicated by the circle, square, triangle, and diamond respectively [insert citations here]. The proposed Parkinson’s Disease mechanistic model may work in conjunction with existing drug-development practices to address scientific goals. Circle: AD amyloid PET: A QSP model was used for prediction of PET change in response to dose (Geerts et al., 2023). Square: Gaucher Disease: Gaucher Disease QSP model used to simulate and optimize the impact of enzyme replacement and substrate reduction treatment protocols (Abrams et al., 2020). Triangle: ALS NFL: Pharmacokinetic model was used for prediction of response to dosing on Tofersen (Paris et al., 2022). Rectangle: Tuberculosis (Hanna et al., 2017). Diamond: References to existing efforts of QSP modeling with respect to the regulatory landscape (Agharmiri et al., 2021; Musuamba et al., 2021).

QSP modeling has been applied to the development of novel therapies across many therapeutic areas, both complex and rare diseases, including cardiovascular and diabetes, immunology and immune-oncology, infectious diseases, and neurological, amongst others (Aghamari et al., 2022). QSP opportunities in the neurosciences are emerging at a rapid pace (Bloomingdale et al., 2021). The impact and range of applications are broad and have historically been on influencing decisions relating to elucidating or advancing the mechanism of action, prioritization of pre-clinical candidates for entry into the clinic, or translational in nature, such as predicting clinical response and leveraging these predictions to optimize clinical design approaches such as selection of biomarkers or endpoints, and response under treatment (Figure 2). Other examples include predicting response of different segments of a clinical population or identifying mechanistic hypotheses to explain differential clinical response to treatment.

Examples of QSP models and their applications include models for cardiovascular diseases that have included building mechanistic and multi-scale dynamical models to predict impact of many novel agents such as sGC activators, neprilysin inhibition, and angiotensinII blockade, PCSK9 inhibition, on cardiac function, systemic circulation and vascular function, blood pressure and lipid metabolism (Ming et al., 2017; Knox et al., 2016; Hallow and Gebremichael, 2017; Peskin and McQueen 1988; Moss et al., 2012). An example in rare diseases is a QSP model for Gaucher disease where the model enabled the prediction of different treatment regimens for maintenance of homeostasis allowing for an optimized and more convenient treatment regimen for patients (Abrams et al., 2020). This is a multi-scale model of Gaucher disease that incorporates calibrated pathways relevant to dysregulation of glycosphingolipid metabolism and specific to cells implicated in the disease process such as splenic macrophages and hepatocytes. Virtual populations of mild to moderate Gaucher disease type-1 were used to simulate the impact of optimizing enzyme replacement and substrate reduction treatment protocols. In ALS, an ALS-SOD1 QSP model which incorporates SOD-1 specific biology and neurofilament light (NFL) biomarker, was developed and used to predict the response to treatment on Tofersen allowing for optimization of dose and treatment protocol (Paris et al., 2022; Biogen, 2023). Moreover, there has been much progress on important QSP model components and building blocks for PD that can facilitate and accelerate the build-up and delivery of QSP application to translational questions, such as an alpha-synuclein model (Righetti et al., 2022). In one mechanistic model, investigators incorporated alpha-synuclein dynamics and other mediators that drive accumulation during disease and potential reduction on treatment. One example of a QSP model with clinical translatability was reported by Roberts et al., which consisted of a detailed biophysical model of a cortico-striatal-thalamic-cortical network for motor symptoms in PD calibrated to the clinical outcome measure Unified Parkinson’s Disease Rating Scale (UPDRS) (Roberts et al., 2016). Such translational models that can be modularized to facilitate a rapid implementation and execution in drug development decision making promise to fill gaps in translatability.

In the context of a defined gene target, such as GBA, LRRK2, or SNCA for Parkinson’s, combining target engagement and modeling tools to define and inform attributes of the translational strategy, such as dose, biomarkers, and patient selection can be an effective strategy in paving a successful path to clinical proof of concept. Combining known mechanistic biomarkers such as alpha-synuclein and pk-pd data for the specific target into a QSP framework, allows drug developers including biologists, clinicians, and modelers to work together in refining the attributes of the translational strategy, increasing the probability of achieving clinical target engagement, and advancing with greater confidence into clinical study. The current dynamic landscape of PD therapeutics provides a unique opportunity and framework for how QSP modeling, when combined with the appropriate data and expertise can be leveraged to increase confidence in efficiently advancing promising DMTs in ways that align with regulatory expectation.

## 4 Overview of existing initiatives and associated databases towards enabling effective and efficient clinical path to proof of concept

The overarching need to address the challenges around successful early clinical investigation and appropriate target engagement leads us to assess available *versus* gaps in data and knowledge. We have seen extensive development of public databases and sources across several institutions and partnerships and look to leverage this rich wealth of knowledge and data, in context and at scale to enable successful and de-risked clinical paths to proof of concept and inform effective and efficient target engagement (Table 1 and Table 2).

**TABLE 1:** Table of Parkinson’s Disease (PD) databanks and their offered data types.

Database	Genomics	Proteomics	Metabolomics	Imaging	Clinical
PPMI	X	X	X	X	X
AMP PD	X	X		X	X
GP2					
PDBP	X	X		X	X
EPND					

**TABLE 2:** Table of Parkinson’s Disease (PD) databanks with studies using measurement in associated biofluids.

Data bank	Plasma	PBMC	Whole blood	CSF	Human IPSCs
PPMI	X	X	X	X	X
AMP PD (GP2)	X	X	X	X	?
PDBP	X	X	X	X	?

### 4.1 Sources of data for PD

Multi-scale data that is grounded in genetics, proteomics, and biomarkers is a true catalyst for analysis of disease progression particularly in heterogeneous disorders including PD. The rapid evolution and scientific advances in combination with big data genomics that can be carried out in millions of individuals worldwide have been transformative and hold true potential in enabling precision medicine strategies (Ou et al., 2021). Biomarkers are key in defining pathophysiologic correlates of clinically meaningful change at various stages of the disease spectrum. There are several examples of complex data of PD observational cohorts that are available to serve as substrate for QSP modeling to support gene-based therapeutic strategies (Klein et al., 2018; Marek et al., 2018; Iwaki et al., 2021; Lange et al., 2023; Vollstedt et al., 2023).

#### 4.1.1 PPMI

The Parkinson’s Progression Marker Initiative (PPMI) sponsored by the Michael J. Fox Foundation is a global collaborative initiative launched over a decade ago aimed to identify and validate biomarkers of onset and disease progression in a multicenter natural history study (Marek et al., 2018). The ultimate goal of PPMI is to improve understanding of disease etiology and course and to provide crucial tools to enhance the likelihood of success of PD modifying therapeutic trials. Critical success factors include alignment and consensus on common data standards for sample and imaging biomarker collection and the rapid integration of all study data into the PPMI database. An independent PPMI biospecimen review committee oversees the biobank and sharing of biological samples including blood, cerebrospinal fluid (CSF), and urine which are made publicly available to scientists.

Open sharing of the data and biological samples with the external community of researchers and trialists has led to seminal discoveries in terms of the underlying biology of PD. PPMI comprises a partnership of government, PD foundations, industries, and academics that work cooperatively together to advance research and therapeutics for PD. To date, the PPMI data has been downloaded and analyzed by investigators across the globe and is a rich data source for industry in designing their clinical trials.

PPMI data has been effectively used for the discovery of novel therapeutic targets, to identify candidate biomarkers for clinical trial decision-making, for formal regulatory endorsement of imaging biomarkers, for clinical trial enrichment, and recently, to enable the measurement of alpha-synuclein in humans at a time up to 8 years prior to onset of clinical diagnosis.

#### 4.1.2 Accelerated medicines partnership for PD (AMP PD)

The PD accelerated medicines partnership was launched in 2018, with the goal of improving clinical trial design and identifying new targets and pathways for therapeutic development. The 5-year effort is funded jointly by NIH and industry and has created a harmonized data platform to support the identification of targets and biomarkers for PD prognosis and disease progression. The AMPPD program has gathered rich clinical and molecular characteristics from PD cohorts. To date, the data is being analyzed by global researchers to discover new targets and biomarkers with focus on modalities such as proteomics, genomics, metabolomics, and other molecular modalities analyzed from biospecimens. A total of eight cohorts have been collected for PD and Dementia with Lew Bodies (DLB) with several publications that have emerged from analysis of the data to date (https://www.amp-pd.org/unified-cohorts). Request for data can be accessed via https://www.amp-pd.org/register-for-amp-pd.

#### 4.1.3 Global Parkinson’s genetics program (GP2)

The monogenic network of GP2 aims to create an efficient infrastructure to accelerate identification of novel genetic causes of PD. A key area of focus is to investigate the underlying mechanisms such as reduced penetrance and variable clinical expression of known disease cause variants. Multidiscipliary experts from around the world are collaborating in prospective ways such as Whole-genome sequencing for up to 10,000 people with Parkinsonism. A recent review (Lange et al., 2023) outlines the workflow and outreach as well as plans for sharing of data in ways that will advance the field overall. Partnerships with relevant consortia are also in place to enhance learnings across public private partnerships. This includes AMPPD, MJFF Global Genetic PD cohort (Vollstedt et al., 2023), and EPND (https://epnd.org/).

#### 4.1.4 Parkinson’s Disease Biomarker Program (PDBP)

The National Institute of Neurological Disorders and Stroke Parkinson’s Disease Biomarker Program (PDBP) was launched following a 2012 workshop that identified gaps in biomarkers for PD. PDBP aims to support PD biomarkers research by leading laboratory and clinically based biomarker discovery for PD. A data management resource (DMR) is in place to support standardization and data sharing from well-characterized longitudinal clinical cohorts, with detailed clinical data collected and biospecimens banked. PDBP serves to align and collaborate with other PD biomarker initiatives such as the Michael J. Fox Foundation (PPMI), whose goal is validation of biomarker discovery projects and BioFIND, an observational cross-sectional study cohort. The inclusion of atypical Parkinson’s and DLB is another unique aspect of PDBP. Numerous publications have emerged from data that is supported by PDBP. A comprehensive review of PDBP was published in 2017 (Gwinn et al., 2017): with much progress emerging over the past years (Chen-Plotkin et al., 2018; Sadaei et al., 2022).

## 5 Advances in regulatory landscape and utility of modeling and simulation for addressing and accelerating drug development regulatory milestones

Global regulatory agencies have recommended Public-Private Partnerships (PPPs) as efficient ways to advance drug development tools. Modeling and simulation drug development tools are important not only in individual drug submissions during formal review of new Investigational New Drugs (INDs) but also pioneering ways to collaborate. Formal regulatory endorsement of modeling tools has been achieved by precompetitive collaboration in a range of diseases including Alzheimer’s and Tuberculosis. The Federal Drug Administration’s (FDA) fit-for-purpose path and the European Medicines Agency’s (EMA) qualification of novel methodologies provide a unique framework for consortia to engage regulators in data-driven ways. Once endorsed, modeling tools are then used to de-risk future targets, according to a defined context use, and serve to streamline regulatory review of new chemical entities (NCEs) oftentimes in ways that are mechanism independent/target agnostic.

Most recently, regulators are recommending expanding Modeling & Simulation (M&S) tools from Ph2/3 decision-making applications to the earlier translational stages of drug development. Physiologically based pharmacokinetic (PBPK) modeling aims to enable integration of physiological, and drug-dependent preclinical and clinical information to model an investigational drug’s absorption, distribution, metabolism, and excretion, predict drug exposure at the site of action, as well as an invaluable tool for predicting drug-drug interactions. The FDA has led PBPK modeling in a variety of different applications including chronic kidney disease and pediatric dose extrapolations (Leong et al., 2012; Hsueh et al., 2018). More recently, the FDA has advocated PBPK and QSP models across a broad range of applications, including guidance documents describing the format and content of PBPK submissions in support of sponsor applications and recent best practices published from a public workshop on PBPK modeling (https://www.fda.gov/regulatory-information/search-fda-guidance-documents/physiologically-based-pharmacokinetic-analyses-format-and-content-guidance-industry, https://www.fda.gov/regulatory-information/search-fda-guidance-documents/use-physiologically-based-pharmacokinetic-analyses-biopharmaceutics-applications-oral-drug-product, https://www.ncbi.nlm.nih.gov/pmc/articles/PMC8592512/) (Center for Drug Evaluation and Research, 2020; Center for Drug Evaluation and Research, 2021; Jean et al., 2021). Translational Model Informed Drug Development (MIDD) strategies are particularly a need where one sponsor is unlikely able to gather sufficient data on their own to enable efficient drug development decision-making needed for clinical trial design (Li et al., 2022). In May 2023, at a QSP MIDD rare disease workshop, FDA leaders recommended that consortia could address gaps that regulators face by carrying out and publishing comprehensive literature reviews on leading disease candidates being advanced by multiple sponsors (https://www.fda.gov/drugs/news-events-human-drugs/creating-roadmap-quantitative-systems-pharmacology-informed-rare-disease-drug-development-05112023). Moreover, a publication summarizing the assessment of QSP models and their application in drug development presented the key challenges and opportunities discussed at an FDA-Industry meeting (Bai et al., 2021; Center for Drug Evaluation and Research, 2023).

A case example of collaborative approaches to advancing MIDD is that of Critical Path Institute. C-Path has the mission of leading multistakeholder collaborations that accelerate drug development, advancing better treatments for people worldwide. A key core competency is in the area of modeling and simulation tools. A unified strategy based on Advancing fit-for-purpose Quantitative Tools to Accelerate Drug Development has been applied across multiple diseases of high unmet medical need. The overall strategy centers on integration of academic natural history patient-level datasets and clinical trial data into a unified data platform. The unified data is then used to develop a quantitative description of disease progression, accounting for relevant sources of variability, including treatment, demographics, genetics and biomarkers, with submission of such models as drug development tools to FDA and EMA for endorsement. Regulators are increasingly reaching out to the broad scientific and patient communities to seek input and recommendations on key regulatory innovative strategies and new frameworks. For example, FDA has released numerous new guidances in 2023 on topics spanning real world data, digital health technologies and artificial intelligence (AI) and machine learning (ML) (https://www.fda.gov/science-research/science-and-research-special-topics/real-world-evidence, https://www.fda.gov/science-research/science-and-research-special-topics/artificial-intelligence-and-machine-learning-aiml-drug-development) (US Food and Drug Administration, 2023).

There are new initiatives coauthored by FDA and EMA aimed to seek harmonization on model informed drug development strategies that includes QSP (Marshall et al., 2023). The community engagement of experts is encouraged to provide input to such efforts to influence and guide the fit for purpose development, implementation, validation, and application of modeling technologies to defined context of use development activities.

Now is the time to advance collaborative modeling strategies for drug development in PD, at a moment in time when the field is experiencing an inflection of knowledge on gene segments, biomarkers, and translational research more broadly.

The list of recommended actions below is aimed at identifying a potential roadmap to success in collaboratively advancing data driven QSP models for PD DMTs.

### 5.1 Call to action for advancing precompetitive QSP models to support precision medicine strategies

• Prioritize gaps in PD therapeutic development focused on advancing gene based therapeutic trials for PD.
• Define context of use for proposed QSP model drug development tool.
• Determine the relevant sources of data that are available and how they will be utilized to support the QSP drug development tool.
  • Data-driven strategies take advantage of the current wealth of relevant data.
  • AMPPD, PPMI, GP2, CPP integrated database, PDBP, tool compounds and associated data
  • Industry agrees to align on providing tool compounds that are shared for proof of pharmacology modeling in agreed upon models (animal, human induced pluripotent stem cells, human).
• Prospective cataloguing of key relevant data sources to demonstrate robustness of QSP model output with independent datasets, under specified contexts of use.
• Multi-stakeholders come together under current PD public private partnerships to collaborate on new PD focused QSP model initiative: industry representatives, regulators, CPATH, nonprofit research organizations, NIH, clinicians, academic PD experts*.
• All stakeholders agree to share costs and data: Sharing of QSP model development and final validated model structure and code, systems wide parameters, tool compounds and data and associated simulations.
• Formal request to regulatory agencies to advance a quantitative drug development tool for review and endorsement via Model informed drug development initiatives: FDA fit for purpose path, EMA qualification of novel methodologies.

*Critical success factors: Regulatory agencies are fully engaged from the genesis of the project and are engaged throughout all stages to provide guidance and recommendations.

The above path has been successfully advanced at Critical Path Institute across many disorders including Alzheimer’s disease, Huntington’s disease, Duchenne Muscular Dystrophy (reviewed by Stephenson et al. (2023), and Type 1 Diabetes (Podichetty et al., 2022). There has been much progress by the Parkinson community to deliver on shared clinical and biomarker databases. In conjunction with additional candidate specific biology and pharmacology datasets, and incorporation into a QSP prediction framework, we can realize the opportunity to advance and de-risk the development of each individual drug candidate and ultimately portfolio of candidates, increasing the probability of approval of a drug for PD on the horizon.

This moment of opportunity, to translate the advances in science and technology, available data, and translational capability and expertise is unprecedented, and provides a unique and exciting leverage to accelerate the development of innovative medicines for Parkinson’s patients. We are confident and eager that this community of patients, regulators, researchers, and sponsors will come together to capitalize on this state of science and opportunity for an incredible mission and cause.

## Data Availability

The original contributions presented in the study are included in the article/Supplementary material, further inquiries can be directed to the corresponding author.
